# Using One-Step Acid Leaching for the Recovering of Coal Gasification Fine Slag as Functional Adsorbents: Preparation and Performance

**DOI:** 10.3390/ijerph191912851

**Published:** 2022-10-07

**Authors:** Tianpeng Li, Shaocang He, Tingting Shen, Jing Sun, Chenxu Sun, Haoqi Pan, Dehai Yu, Wenxue Lu, Runyao Li, Enshan Zhang, Xuqian Lu, Yuxuan Fan, Guiyue Gao

**Affiliations:** 1 School of Environmental Science and Engineering, State Key Laboratory of Biobased Material and Green Papermaking, Qilu University of Technology (Shandong Academy of Sciences), Jinan 250353, China; 2 Yankuang National Engineering Research Center of Coal Water Slurry Gasification and Coal Chemical Industry Co., Ltd., Jinan 250000, China

**Keywords:** solid waste reutilization, coal gasification fine slag, acid leaching, adsorption mechanisms, wastewater treatment

## Abstract

Coal gasification fine slag (FS), a kind of by-product of coal chemical industry, was recovered for the preparation of functional adsorbents by acid leaching process, which was orthogonally optimized by HCl, HNO_3_, HF, HAc, and H_2_SO_4_. Methylene blue (MB) was used to evaluate the performance of functional adsorbents. The results demonstrated that 57.6% of the leaching efficiency (R_LE_) and 162.94 mg/g of adsorption capacity (C_AC_) of MB were achieved under the optimal conditions of HNO_3_ of 2.0 mol/L, acid leaching time of 2.0 h, and acid leaching temperature of 293K. The detections on X-ray Diffraction (XRD), Scanning Electron Microscope (SEM), Fourier Transform Infrared Spectroscopy (FTIR), and BET surface area (SBET) indicated that the synthesized functional adsorbents were characterized by mesoporous materials. The good fitting of adsorption process using pseudo-second-order and Langmuir models demonstrated that the chemisorption contributed to MB removal. The results of thermodynamics further revealed that the adsorption process of MB occurred spontaneously due to the exothermic properties. The work is expected to develop a novel and cost-effective strategy for the safe disposal of FS, and potentially offer an alternative pathway to increase the additional value for the coal chemical industry.

## 1. Introduction

China’s energy status quo is characterized by lean oil and rich coal. To make better use of abundant coal resources, coal gasification technology is introduced to produce methane, hydrogen, and other combustible gases through the gasification of coal water slurry [1]. Coal gasification slag (CGS) is the by-product of coal gasification technology. The large accumulation of coal gasification slag would lead to the wastage of soil resources, cause pollution of air, water, and soil, and endanger human health [2]. The resource utilization of CGS has been an inevitable topic in the development process of the coal gasification industry.

At present, CGS is mainly reutilized in the fields of industrial production, agricultural industry, building materials, energy recovery, and environmental protection. The industrial application is mainly to add CGS to rubber and plastic, which increases the properties of the polymer [3,4,5,6]. In agriculture, CGS is mainly used as a soil conditioner [7], and a source of silicon fertilizer [8] to improve the composting effect [9,10]. In construction, CGS can be mixed with cement to modify the performance of cement [11], fire bricks [12], and backfill mines [13]. As for energy recovery, CGS can be used to prepare fuel [14,15], used as a battery [16], burned into carbon [17] due to the constituent of residual carbon.

Recently, the application of CGS in pollutant treatment and disposal is mainly used as foundational materials. Liu et al. [18] leached CGS with HCl and removed carbon in a Muffle furnace to obtain a kind of mesoporous material. Zhao et al. [19] found that FS had good adsorption performance on water distribution and adsorption behaviors. due to the pore structure. Additionally, the preparation of zeolite materials from CGS increased great concern due to the pore structure of zeolite materials being more orderly and controllable. There are several types of existing CGS zeolite materials, including single-phase A zeolite [20], NaP zeolite [21], MCM-41 zeolite [22,23], and ZSM-5 zeolite [24]. The common characteristics of the above investigations are involved in the energy input due to the high reaction temperature, which might increase the cost of production.

Actually, CGS could be divided into two kinds of slags, including coarse slag (CS) and fine slag (FS) according to coal gasification process. FS contains a lot of unreacted carbon and a small number of metal substances, which is superior to that of CS, so FS is often recovered as a functional adsorbent to remove pollutants, such as adsorption of dyes [18,25], heavy metals [26], deodorization, and flavor removal [27,28], microwave absorption [29], carbon capture, and transportation [30,31]. Recently, researchers have investigated that MB, as a toxic organic dye, caused water eutrophication, stimulated the human digestive tract, and caused ecological damage [32]. Removal of MB is conducive to maintaining ecological balance and protecting human health. On the other hand, MB has good color rendering; it is very convenient to observe its adsorption process and build the adsorption model. MB is a very common adsorbate in the adsorption process and has a great reference value [33].

Based on the above research, it could be deduced that the reutilization of CGS is an emerging crucial task in the field of the coal chemical industry, and the cost-effective strategy of reducing, recycling, and reusing is of great importance. Therefore, in this work, a one-step leaching process was developed for the preparation of functional adsorbents, and the performance was evaluated by MB removal. Five kinds of acids, including HCl, HNO_3_, HF, HAc, and H_2_SO_4_, were used to optimize the leaching process, which was investigated by the treatment of MB with single-factor and orthogonal experiments. Furthermore, the adsorption mechanisms of functional adsorbents were investigated by adsorption kinetics, adsorption thermodynamics, and adsorption isotherm. It is expected to provide a novel and cost-effective strategy for the reutilization of CGS and improve the additional value of coal chemical industry.

## 2. Materials and Methods

### 2.1. Materials

The FS was provided by Yankuang National Engineering Research Center of Coal Water Slurry Gasification and Coal Chemical Industry Co., Ltd. The powder of FS was screened and its particle size was less than 150 μm. HCl and H_2_SO_4_ used in the experiment were purchased from Yantai Yuandong Science and Technology Co., Ltd., Yantai, China. HNO_3_, HF, HAc, potassium hydrogen phthalate, potassium fluoride, potassium chloride, ethanol, sodium hydroxide, ammonia, potassium hydroxide, calcium carbonate, EDTA, sodium acetate, copper sulfate, and ammonium triacetate were all purchased from Shanghai Pharmaceutical Co., Ltd., Shanghai, China, and phenolphthalein, calcein, xanthyl salicylic acid, and MB were purchased from Tianjin Kemiou. DL-mandelic acid and PAN were purchased from McLean. All the reagents used in the experiment were of AR. The above five acids were prepared as a stock solution (10.0 mol/L) and diluted to designed concentration for acid leaching.

### 2.2. Acid Leaching Process Optimization

All acid treatment reactions except for HF were carried out in 250 mL glass beakers, and HF reaction was conducted in 300 mL plastic beakers in water baths. The samples were filtered and repeatedly washed with deionized water until the solution was neutral, then placed in the electrothermal blowing dry box drying at 333 K for 24 h. The calculation formula for leaching efficiency (R_LE_) was shown in Formula (1). The content of inorganic components (Si, Fe, Al, Ca) was determined by chemical analysis. The calculation formula of metal element leaching was according to Formula (2). The sample number was named with FS by acid type, acid concentration, leaching time, and temperature, and HCl, HNO_3_, H_2_SO_4_, HAc, and HF were represented by H, N, S, C, and F respectively. For example, FS-N-2M-2 h-293K was denoted as the sample obtained by 2.0 mol/L HNO_3_ with acid leaching time of 2 h and leaching temperature of 293 K. The ratio of slag to liquid was constant at 1:10 (*m*/*v*), and the stirring rate was 120 r/min.

(1) $$R_{LE}=\frac{m_{e}-m_{0}}{m_{L}}\times 100\%$$

here *R_LE_* represents the leaching efficiency of FS, *m*_0_ represents the weight of filter paper plus beaker before filtering, *m_e_* represents the weight of beaker plus filter paper plus sample after drying filter residue, and *m_L_* represents the weight of raw materials added in the leaching process of FS.

(2) $$R_{LEM}=\frac{C_{m}V_{L}}{m_{L}W_{XRF}}\times 100\%$$

where *R_LEM_* represents the leaching efficiency of metal, *C_m_* is the concentration of metal ions in the solution, which is calculated by DLT 1037-2016. *V_L_* is the volume of solution in the acid leaching process, which is calculated by adding the volume of the acid solution. *W_XRF_* represents the mass fraction of metal material obtained by *XRF* analysis (Table 1).

In addition to the normal control experiment, an orthogonal experiment of *L*_16_(4^4^) was designed to further optimize the operational conditions (Table S1). Through orthogonal analysis, the relationship between leaching effect and adsorption performance was explored.

### 2.3. Characterization

For the characterization, the original FS and the samples leached with 2.0 mol/L HCl, HNO_3_, H_2_SO_4_, HAc, and HF at the temperature of 293 K for 2.0 h.

The crystal lattice structure and crystallinity of the material were analyzed by X-ray Diffraction (XRD, D8ADVANCE, Karlsruhe, Germany). Radiation waves were Cu-Kα (λ = 1.5418 A) at 40 kV, 40 mA, deflection angle 2θ = 5–80°. The surface morphology of the sample was observed by scanning electron microscope (SEM, Regulus 8220, Japan), and the elemental composition of the sample was observed by EDS. The composition of surface functional groups was analyzed by Fourier transform infrared spectroscopy (FTIR, Nicolet iS 10, USA). X-ray fluorescence (XRF, PANalytical Axios, Holland, The Netherlands) was used to analyze the composition and content of elements in the original samples. The specific surface area (SSA) was analyzed by Anton Paconta SBET analyzer (Autosorb-IQ, Graz, Austria). Nitrogen was injected into the instrument and tested at 77 K, and the fitting analysis of the specific surface area, pore diameter, and pore volume measurements was carried out by an ASiQwin software. The concentration of MB was conducted on a UV-visible spectrophotometer at 664 nm (UV-Vis DRS, UV-2550, Shimadzu, Kyushu, Japan). 

### 2.4. Adsorption Mechanisms Investigation

#### 2.4.1. Adsorption Process

In the adsorption experiment, the basic conditions were investigated as 200.0 mL of 100.0 mg/L MB, functional adsorbents of 0.1 g, pH of 9.0, stirred in a 120 r/min, reaction time of 24 h. FS-N-2M-2 h-293K was selected to analyze the adsorption mechanism. The adsorption capacity (C_AC_) of MB, *Q_e_* (mg/g), was calculated by the Formula (3):

(3) $$Q_{e}=\frac{\left(C_{0}-C_{e}\right)V}{W}$$

here *C*_0_ (mg/L) and *C_e_* (mg/L) represent the concentration of MB solution at the beginning and ending respectively; *V*(L) represents the volume of MB solution; *W*(g) represents the mass of functional adsorbent added to the solution.

#### 2.4.2. Adsorption Kinetics

The sample was put into a 250 mL beaker, 200 mL MB of 100 mg/L, stirred in a 120 r/min agitator, and sampled at an interval of 10 min to draw the adsorption kinetics curve. To make validate the adsorption process, three kinetic models, the pseudo-first-order, the pseudo-second-order, and Weber-Morris particle intra diffusion models were used to describe the kinetics of MB adsorption. The relevant equations of Formula (4) (pseudo- first-order model), Formula (5) (pseudo-second-order model), and Formula (6) (Weber-Morris intra diffusion model) were introduced as follows:

Pseudo-first-order kinetic model:

(4) $$Q_{t}=Q_{e}\left(1-e^{-k_{1}t}\right)$$

Pseudo-second-order kinetic model:

(5) $$Q_{t}=\frac{k_{2}Q_{e}^{2}t}{1+k_{2}Q_{e}t}$$

Weber-Morris particle intra diffusion model:

(6) $$Q_{t}=k_{ip}t^{\frac{1}{2}}+C_{i}$$

where *t* (min) represents the reaction time; *k*_1_ (min^−1^) is the pseudo-first-order adsorption rate constant; *k*_2_ (g/mg · min) is the pseudo-second-order adsorption rate constant; *Q_t_*(mg/g) represents the adsorption capacity of the synthesized functional adsorbents at time t (min); *k_ip_* (mg/g·min) is the rate constant of the Weber-Morris intra-particle diffusion model; *C_i_* (mg/g) is a constant related to boundary thickness.

#### 2.4.3. Adsorption Isotherm

Add 200 mL methylene blue solution, whose concentration was 10.0 mg/L–300.0 mg/L, into a 250 mL beaker. The solution is stirred on a coagulation stirrer. Samples were taken at an interval of 30 min until the adsorption reaction reached equilibrium. In the work, three typical isotherm adsorption models of Langmuir, Freundlich, and Temkin were applied to fit the isothermal adsorption data. The relevant equations are expressed in Formulas (7)–(10):

Langmuir isothermal model,

(7) $$Q_{e}=\frac{Q_{\max}K_{L}C_{e}}{1+K_{L}C_{e}}$$

(8) $$R_{L}=\frac{1}{1+K_{L}C_{0}}$$

Freundlich isothermal model,

(9) $$Q_{e}=K_{F}C_{e}^{\frac{1}{n}}$$

Temkin isothermal model,

(10) $$Q_{e}=\frac{RT}{b}ln\left(K_{T}C_{e}\right)$$

*K_L_* (L/mg), *K_F_* (mg/g), and *K_T_* (L/mg) are Langmuir constant, Freundlich constant, and Temkin constant, respectively. *Q*_max_ is the maximum adsorption capacity, *R_L_* is the adsorption isothermal coefficient, *n* is a constant, R = 8.314 J/mol·K is the ideal gas constant, *T* (K) is the absolute temperature, and *b* (J/mol) is the coefficient of thermal effect.

#### 2.4.4. Adsorption Thermodynamics

The adsorption isotherm drawing experiments were repeated at 293 K, 303 K, and 313 K to fit the adsorption thermodynamic model. The free energy change (Δ*G*), enthalpy change (Δ*H*), and entropy change (Δ*S*) are shown in Formulas (11)–(14).

(11) ΔG=ΔH−TΔS

(12) $$\Delta G=-R\cdot T\cdot \ln K_{c}$$

(13) $$K_{c}=\frac{Q_{e}}{C_{e}}$$

(14) $$\ln K_{c}=\frac{\Delta S}{R}-\frac{\Delta H}{RT}$$

where *K_c_* (L/g) equilibrium adsorption coefficient; Δ*G* (kJ/mol) is Gibbs free energy, Δ*S* (kJ/mol · K) is entropy change; Δ*H* (kJ/mol) is the change in enthalpy.

### 2.5. Analysis of Data

The control test adopts a multivariate analysis of variance to understand the analysis of time, concentration, and temperature on the structure of *R_LE_* and C_AC_, and involves an orthogonal experiment, which was combined to find the experimental conditions to achieve the best effect [34]. Through the establishment of an unsaturated model and mean comparison analysis, this work found the most suitable model.

Correlation analysis between *R_LE_* and C_AC_, Kendall τ, and Pearson correlation coefficient [35] were calculated.

## 3. Results and Discussion

### 3.1. Acid Leaching Process Optimization

#### 3.1.1. Acid Type Effect

**Figure 1:**
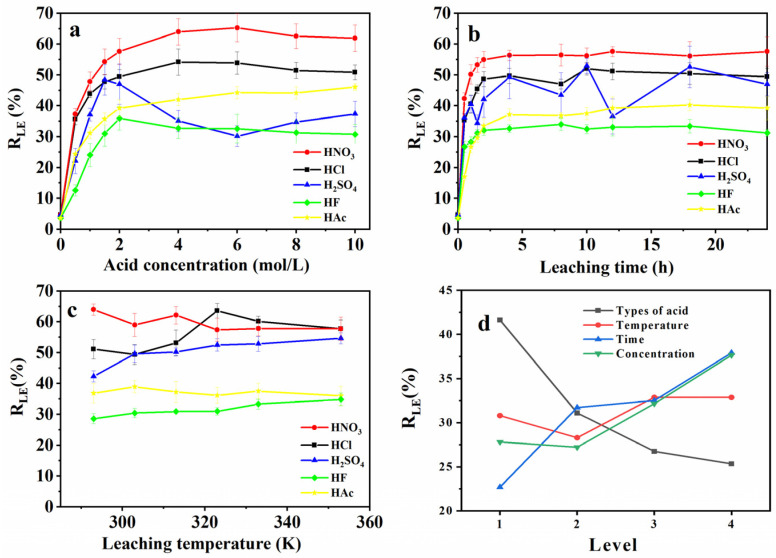
Optimization of acid leaching process. (**a**) acid concentrations, (**b**) leaching time, (**c**) leaching temperature, (**d**) orthogonal level analysis.

FS was treated with HNO_3_, HCl, H_2_SO_4,_ HAc, and HF, and the results were demonstrated in Figure 1. It was found that the R_LE_ of FS decreased in the following way: HNO_3_ > HCl > H_2_SO_4_ > Hac > HF. This indicated that the *R_LE_* of FS treated by HNO_3_, HCl, and H_2_SO_4_ was better than that of the HAc and HF. The acid leaching process was used to remove metal substances from FS, leaving the porous structure for the preparation of the foundation adsorbent. As shown in Figures S1–S3, Ca, Al, Fe, and Si were detected in the leaching solution, and the results could be attributed that the original glass microspheres in the solution and the glass microspheres leached by the acid leaching reaction will partially penetrate the filter paper and enter the filtrate. In addition, there were silicon and metal compounds, and acid leaching caused the silicon to react with the metal to form H_2_SiO_3_ (Formula (15)), which was filtered into the filtrate [36]. The silicon content in the filtrate came from glass microspheres, which generally reflected the modification effect of carbon materials.

(15) $$H^{+}+X_{a}SiO_{b}\to H_{2}O+H_{2}SiO_{3}+X^{\frac{2b}{a}}$$

where *H*^+^ is hydrogen ion, *X* represents metal element, *a* is the number of metal atoms in silicate, *Si* represents the element of silicon, *O* represents the element of oxygen, and *b* is the number of oxygen atom in the silicate.

The results further indicated that the *R_LE_* of HNO_3_ and HCl was better than that of H_2_SO_4_, and HNO_3_ has the highest *R_LE_*. In the metal leaching efficiency diagrams (Figures S1–S3), there is no obvious difference in the leaching ability of metal oxide between HNO_3_ and HCl, except for the leaching rate of silicon. Due to the strong oxidizing property of HNO_3_, it had a stronger ability to modify the carbon surface [37]. *R_LE_* of H_2_SO_4_ was worse than that of HNO_3_ and HCl. The result demonstrated that Ca was often added as a catalyst component in coal gasification production [38], and H_2_SO_4_ reacts with Ca^2+^ forming CaSO_4_, which is insoluble in water. CaSO_4_ blocks the pore and hinders the progress of the reaction.

The ionization degree of HAc and HF was weaker than that of strong acid, leading to the ability of leaching metal. Moreover, F^−^ could react with metal and form CaF_2_, AlF_3_, and FeF_3,_ which might deposit on the surface of the sample, and affect the *R_LE_* and C_AC_ [39]. Additionally, HAc was weak in acidity, and had poor reactivity with Al, inhibiting its *R_LE_*.

#### 3.1.2. Acid Concentration Effect

As shown in Figure 1a, *R_LE_* gradually increased with the increase of acid concentration until it reached equilibrium, and the *R_LE_* of inorganic substances changed with the concentration (Figure S1).

In the high concentration range, the acids exhibited different *R_LE_*. When the concentration reached more than 6.0 mol/L, the *R_LE_* of HNO_3_ decreased. From the analysis of the optimal reaction conditions, the FS can be disposed of by HNO_3_, and the best *R_LE_* can be achieved at 6.0 mol/L. As the acid concentration increased, the reaction between concentrated HNO_3_ and organic matter was no longer oxidation, but a substitution reaction occurred, and the functional groups on the surface of the FS were replaced with amino group [40]. HCl reached the optimal leaching amount at 4.0 mol/L, and the leaching rate curve of silicon in HCl (Figure S1 Si), was consistent with the *R_LE_* curve. The ability of HCl to modify the organic components of the FS was the best. The optimal *R_LE_* of H_2_SO_4_ was 1.5 mol/L. As the concentration of H_2_SO_4_ increased, the concentration of SO_4_^2−^ in the solution increased, promoting the formation of CaSO_4_ precipitation, and reducing *R_LE_*. The HAc leaching of various metal substances was mainly driven by pH, and the increase of HAc concentration promotes the reaction [41]. HF and H_2_SO_4_ showed the same performance. As the concentration increased, insoluble matter precipitated, reducing the *R_LE_*.

#### 3.1.3. Leaching Time Effect

Figure 1b indicated the leaching time effect on *R_LE_*_,_ which could be divided into two stages. In the first stage, as the reaction progresses, *R_LE_* continued to increase, and metal oxides were continuously precipitated (Figure S2). In the second stage, the reaction reached equilibrium. *R_LE_* obtained in the first stage had the same trend, and was different in the second stage.

HNO_3_ and HCl, with the progress of the reaction, kept the *R_LE_* improving. After the reaction reached equilibrium, Fe^2+^, Al^3+^, and H_2_SiO_3_ in the solution formed polymeric aluminum silicate flocculants, which hindered the reaction process (Figure S2), and reduced *R_LE_*. *R_LE_* of H_2_SO_4_ was controlled by the CaSO_4_ content in the system. During the reaction process, after *R_LE_* reached the maximum, the *R_LE_* of CaSO_4_ was constantly changing until it was balanced. In Figure S2, the change of Ca controls the *R_LE_*, and the CaSO_4_ in the H_2_SO_4_ leaching process did not affect the leaching of other metal elements and silicon. HAc was especially weak in acidity, and the reaction process slowed down as the reaction went on, the metal oxides were continuously leached out, and *R_LE_* of FS gradually increased until equilibrium (Figure S2).

#### 3.1.4. Leaching Temperature Effect

As shown in Figure S3, Si appeared in the solution in the form of H_2_SiO_3_ with the rising temperature under acidic conditions, which hindered the progress of the reaction, and reduced the *R_LE_* of HNO_3_ (Figure S3). With the increase in temperature and the break of metal chemical bonds (Figure S3), the *R_LE_* of HCl increased [28]. During the leaching process, the solubility of CaSO_4_ increased with the increase in temperature (Figure S3 Ca), and the content of Ca in the leaching solution continued to increase, so the *R_LE_* of H_2_SO_4_ improved with the increase of temperature. It seemed that the temperature had little effect on the *R_LE_* of HAc, however, higher temperature made the reaction between HF and Si more thorough (Figure S3 Si), and improved the *R_LE_* of HF [42].

### 3.2. Optimization of Leaching Conditions

**Figure 2:**
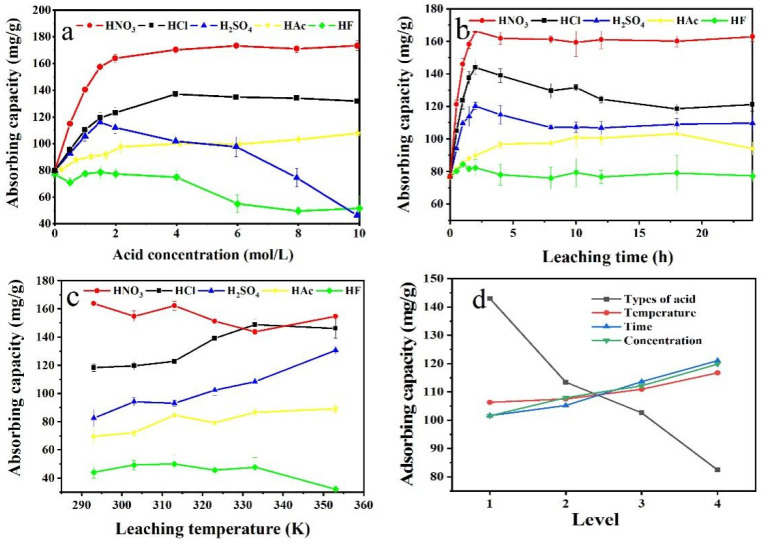
Absorption capacity investigation. (**a**) acid concentration, (**b**) leaching time, (**c**) leaching temperature, (**d**) orthogonal level analysis.

Absorption capacity investigation was conducted in Figure 2, it was found that the C_AC_ of MB was linearly correlated with the *R_LE_* (Table S2). Among them, HNO_3_, HCl, and HAc had a strong correlation due to the higher coefficient, which was much less for HF and H_2_SO_4_. The results could be ascribed that the sedimentation such as CaF_2_ and CaSO_4_ was settled on the surface of the functional adsorbents leached by HF and H_2_SO_4_, which might block the porous structure, decreasing its adsorption effect. Table 2 further indicated that the pore structure improved after the acid leaching because the inorganic components were dissolved out and the rich porous structure was left (Table 2), which played a great role in adsorption capacity.

The results of the multivariate analysis of variance were shown in Table S3. It could be found that most of the correlations R^2^ were more than 0.9500, except for that of C_AC_ of HF, and the Type III Sum of Squares based on *R_LE_* and C_AC_ were in line with leaching time > acid concentration > acid type > temperature. In the orthogonal experimental, *R_LE_* and C_AC_, the Delta conformed acid type > leaching time > acid concentration > temperature. According to the results of the single-factor experiment (Table S3) and orthogonal analysis (Tables S1, S4 and S5, Figure 1d and Figure 2d), the effect of different reaction conditions was in the order of acid type > leaching time > acid concentration > temperature. Based on the above investigation and cost, the optimal condition was selected as 2.0 mol/L HNO_3_ at 293 K for 2.0 h. Under this condition, the C_AC_ was up to 162.94 mg/g while the *R_LE_* was 57.6%.

### 3.3. Characterization of Functional Adsorbents

**Figure 3:**
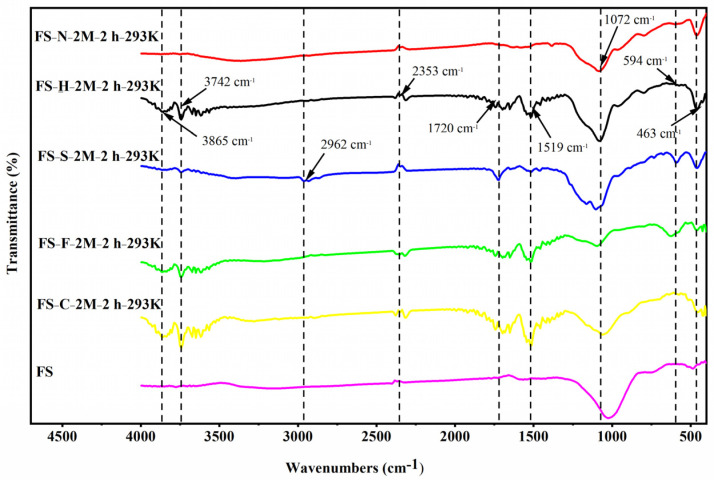
FTIR spectra of initial FS and functional adsorbents.

Figure 3 showed the FTIR spectrum of functional adsorbents. O-Si-O bending vibration peak was generated at 463 cm^−1^ [43]. Si-O-Si or Si-O at 1072 cm^−1^ [44] acted together with carbohydrates to produce the characteristic peak of stretching vibration. The vibration peak at 1519 cm^−1^ was responsible for the HO-H distortion vibration of adsorbed water molecules. The stretching vibration of 2353 cm^−1^ and 1720 cm^−1^ could assign to C=O, and 2962 cm^−1^ might attribute to the asymmetric vibration of C-H [45], and the bending vibration of -OH might be located at 3865 cm^−1^ and 3742 cm^−1^. Functional adsorbents leached by HCl, HF, HAc, and H_2_SO_4_ occurred new functional groups, whereas those treated with HNO_3_ did not generate new functional groups.

**Figure 4:**
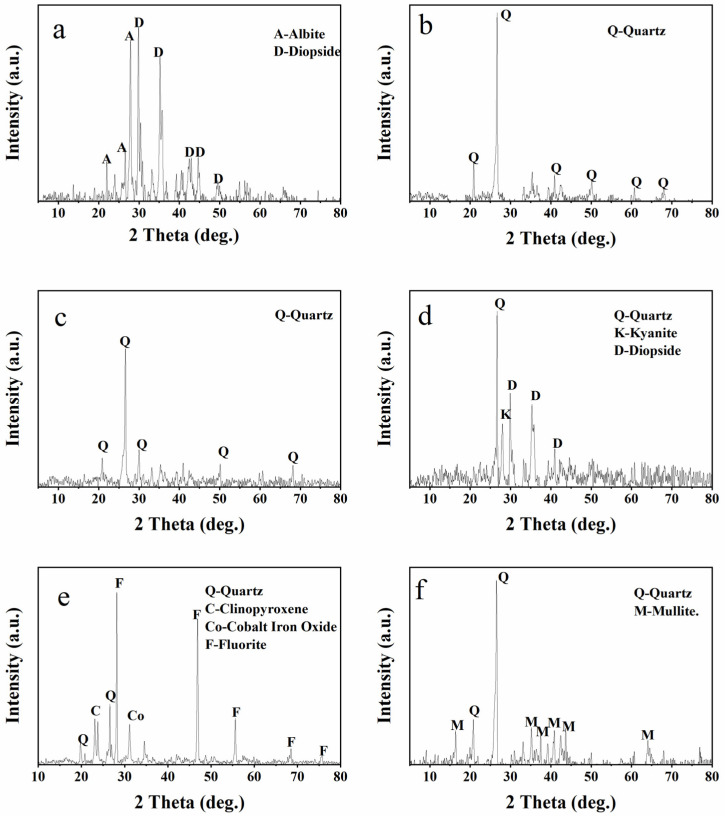
XRD analysis of initial FS and functional adsorbents after combustion. (**a**) initial FS, (**b**) FS-N-2M-2 h-293K, (**c**) FS-H-2M-2 h-293K, (**d**) FS-S-2M-2 h-293K, (**e**) FS-F-2M-2 h-293K, (**f**) FS-C-2M-2 h-293K.

The XRD of the functional adsorbents can be divided into two categories. The functional adsorbents were directly leached by acid (Figure S4), and those obtained after acid leaching were burned in a muffle furnace at 1123 K for 3 h (Figure 4). In Figure S4, FS and functional adsorbents treated with HCl, HNO_3_, and HAc showed peaks in the quartz phase. The functional adsorbents treated with H_2_SO_4_ showed the structure of CaSO_4_ (Figure S4e), and those treated with HF occurred in SiO_2_ and fluoride crystal phases (Figure S4f). The results showed that disordered albite and thermo-refractive glycoside crystal phases appeared in FS after combustion (Figure 4a), and only the quartz crystal remained in the burned functional adsorbents treated with HCl and HNO_3_ (Figure 4b,c). Before and after burning, the peaks produced by the functional adsorbents treated with H_2_SO_4_ and HF (Figure 4d,e) had no significant change. The mullite crystal phase was produced in the functional adsorbent treated with HAc (Figure 4f), and aluminum was not completely removed during acid leaching.

**Figure 5:**
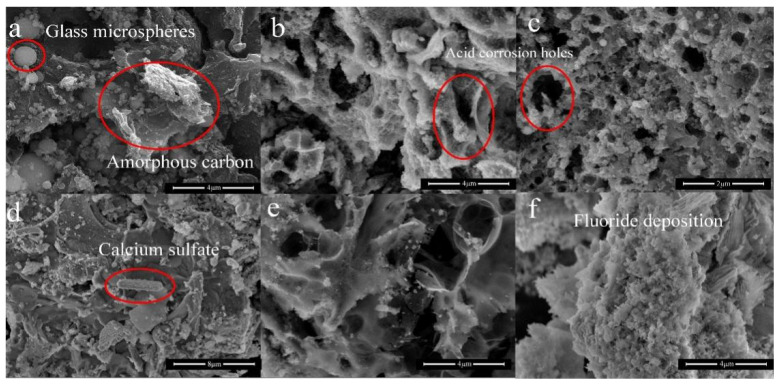
SEM of initial FS and functional adsorbents. (**a**) initial FS, (**b**) FS-N-2M-2 h-293K, (**c**) FS-H-2M-2 h-293K, (**d**) FS-S-2M-2 h-293K, (**e**) FS-C-2M-2 h-293K, (**f**) FS-F-2M-2 h-293K.

The SEM of FS and the functional adsorbents were shown in Figure 5. It could be seen that FS was composed of molten glass microspheres and amorphous carbon (Figure 5a). The glass microspheres were divided into two parts, one part existed alone, and the other part was wrapped in carbon. Figure 5b,c was the functional adsorbents leached by HNO_3_ and HCl, which are characterized by bigger pores, the basic skeleton of the surface, and the three-dimensional pore structure that had not changed [11], and the surface of HNO_3_ treated functional adsorbents was smoother. Furthermore, it was obvious that functional adsorbents synthesized by H_2_SO_4_ leaching process, the surface of which was covered with a layer of CaSO_4_ particles (Figure 5d), and which was slightly corroded with larger porous structure in HAc leaching process (Figure 5e), and the surface of functional adsorbents synthesized by HF was the roughest one due to deposition of fluoride (Figure 5f). The results further demonstrated that the acid type played a crucial role in the leaching process. Additionally, combined with Figure 5e,f, Figures S5 and S6, it could be deduced that the content of metal and silicon in functional adsorbents decreased, leading to the improvement of the porous structure.

**Figure 6:**
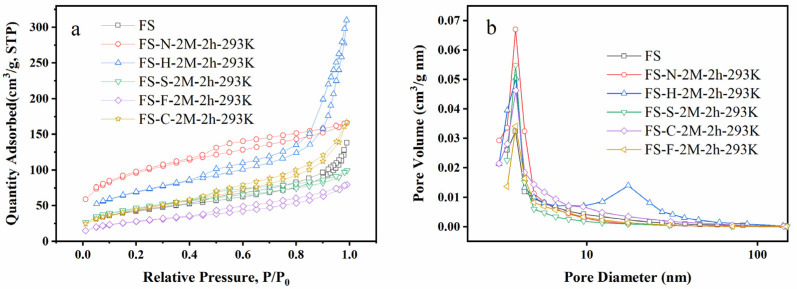
(**a**) N-adsorption/desorption isotherms of initial FS and functional adsorbents, (**b**) Pore size distribution of initial FS and functional adsorbents.

In the N_2_ adsorption and desorption curves in Figure 6a, except for the functional adsorbents leached with HNO_3_, the isotherms during the adsorption process were all type IV adsorption isotherms, and the functional adsorbents treated with HNO_3_ showed the characteristics of type II adsorption isotherms, which proved that the pore structure of the functional adsorbents after HNO_3_ treatment played a great role in the improvement of adsorption capacity. H3-type hysteresis loops appeared in all functional adsorbents with complex pore structures. In the pore size distribution curve (Figure 6b), the pores were mainly distributed in the mesoporous region, and FS-H-2M-2 h-293K had a certain pore size distribution in the macropore range, the results were in agreement with that of SEM. Table 2 showed that the pore size changed from 19.09 nm to 3.82–3.83 nm after acid leaching. The specific surface area increased from 151.67 m^2^/g to 340.40 m^2^/g, and the pore volume increased from 0.21 cc/g to 0.48 cc/g. The results firmly proved that the functional adsorbents were characterized by mesoporous materials.

### 3.4. Adsorption Mechanisms Investigation

The adsorption kinetic model was investigated by pseudo-first-order kinetics, pseudo-second-order kinetics, and the Weber-Morris model [46,47], and the adsorption isotherm was depicted by Langmuir adsorption isotherm, Freundlich adsorption isotherm, and Temkin adsorption isotherm model [48,49]. Furthermore, the adsorption thermodynamic model [50] was demonstrated by Gibbs free energy (Δ*G*), entropy change (Δ*S*), and enthalpy change (Δ*H*).

#### 3.4.1. Adsorption Kinetics

**Figure 7:**
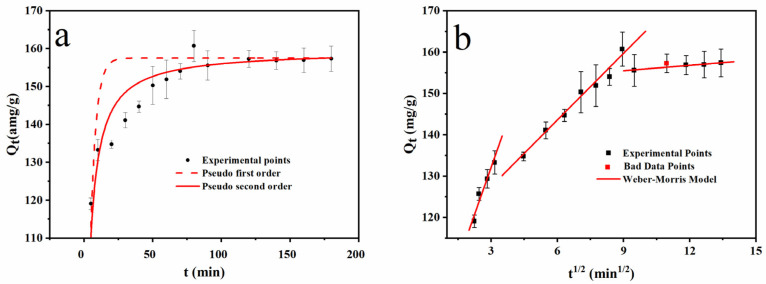
Kinetics investigation on FS-N-2M-2 h-293K. (**a**) pseudo-first-order and pseudo-second-order; (**b**) Weber-Morris Model.

As shown in Figure 7a and Table 3, the adsorption process was in line with that of pseudo-second-order kinetic model due to the correlation coefficient R^2^ of 0.875 and χ^2^ of 12.193. The theoretical saturated adsorption capacity was 156.10 mg/g, which showed the relative error within 5% compared with 153.94 mg/g at 200.0 min, and the results were in line with expectations. Moreover, the fitting of the Weber-Morris model (Figure 7b) showed that the adsorption process was divided into three stages. The curves of the three stages were all beyond the origin, indicating that the adsorption process was not only limited by the internal diffusion of the adsorbate particles, but also limited by other factors. The first section of the curve was the mass transfer process of MB outside FS-N-2M-2 h-294K. The second section was the mass transfer process of MB molecules in the inner pores of the functional adsorbent. The third section indicated the adsorption equilibrium. The diffusion rate constant K_i1_ > K_i2_ > K_i3_ (Table 3) demonstrated that the external mass transfer rate of the adsorbate molecules was very fast during the adsorption process. After crossing the liquid film on the surface of FS-N-2M-2 h-294K, it entered the u-empty-island in the functional adsorbent lowly into the internal pores of the particle.

#### 3.4.2. Adsorption Isotherm

**Figure 8:**
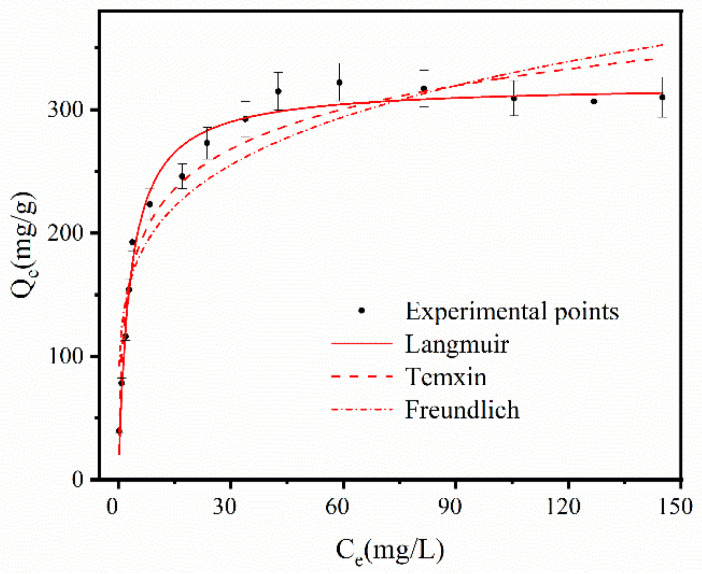
Adsorption isotherm investigation on FS-N-2M-2 h-293K at room temperature.

The adsorption isotherm of MB in Figure 8 was measured by changing the concentration of MB in the solution, and Table 4 was the coefficient of adsorption isotherm at different temperatures. It can be seen that the correlation coefficient Langmuir > Temkin > Freundlich, Langmuir was greater than 0.9 at different concentrations, and the fitness was the highest among the three adsorption isotherms, indicating that the monolayer adsorption occurred on the surface of the FS-N-2M-2 h-294K for MB. 0 < R_L_ < 1, and 1 < n in Freundlich, it can be judged that preferential adsorption occurred in the reaction. It was proved by the Temkin model that there was a strong molecular force in the adsorption process. The good fitting of adsorption process of pseudo-second-order and Langmuir models demonstrated that MB adsorption was involved in the chemisorption rate controlling mechanism.

#### 3.4.3. Adsorption Thermodynamics

In Table 5, Δ*G* was negative at different temperature, indicating that the reaction could proceed spontaneously, the absolute value of Δ*G* decreased with the increase in temperature, and the adsorption driving force of MB was stronger at a lower temperature. During the adsorption process, Δ*S* was less than zero, indicating that the whole system was more orderly after the MB were adsorbed on the surface of the FS-N-2M-2 h-294K during the adsorption process. Δ*H* less than zero proved that the adsorption process of MB was exothermic.

Furthermore, a performance comparison of adsorption effect on MB adsorption between the resulted functional adsorbent FS-N-2M-2 h-293K and previously reported adsorbents has been depicted in Table 6 [51,52,53,54,55]. The results of the analyses revealed that the novel functional adsorbent gains advantage over many other adsorbents, indicating that FS-N-2M-2 h-293K is a potential promising strategy for the treatment of MB wastewater. Simultaneously, it could be further proved that the acid leaching process was an effective way for the reutilization of FS.

## 4. Conclusions

The work successfully initiated one-step process of acid leaching for the preparation of functional adsorbent based on FS without extra chemical reagents and energy input. Moreover, the effluent from acid leaching could be further recycled for the neutralization of industrial alkaline wastewater, and the filtration from acid leaching could be further applied to prepare multi-ionic flocculants. Therefore, one-step acid leaching was characterized by cost-effectiveness, high efficiency, easy operation, and less occupied area. The work provided a safe way for economically viable production of adsorbents, and simultaneously offered a novel strategy for increasing the additional value of coal chemical industry.

## Figures and Tables

**Table 1:** XRF analysis of FS (wt.%).

	Si	Al	Fe	Ca	Na	Mg	K	S	Ti	Others	LOI
FS	21.20	11.15	13.90	13.44	1.39	1.22	1.06	0.82	0.70	1.47	33.68

LOI: Loss On Ignition.

**Table 2:** The pore properties of functional adsorbents.

	Pore Diameter (nm)	Total Pore Volume (cc/g)	Surface Area (m^2^/g)
FS	19.09	0.21	151.67
FS-N-2M-2 h-293K	3.82	0.26	340.40
FS-H-2M-2 h-293K	3.83	0.48	247.21
FS-S-2M-2 h-293K	3.82	0.15	162.09
FS-C-2M-2 h-293K	3.83	0.26	159.01
FS-F-2M-2 h-293K	3.83	0.12	98.83

**Table 3:** Kinetics analysis of FS-N-2M-2 h-293K.

Kinetics Model		Parameters			
Pseudo-first-order		Q_e_	K_1_	R^2^	χ^2^
		156.10	0.26784	0.523	46.665
Pseudo-second-order		Q_e_	K_2_	R^2^	χ^2^
		159.47	0.00282	0.875	12.193
Weber-Morris model	Part I	C_i_	K_i1_	R^2^	χ^2^
		86.57022	15.18055	0.90365	2.65117
	Part II	c	K_i2_	R2	χ^2^
		111.009	5.381	0.979	9.32794
	Part III	c	K_i3_	R2	χ^2^
		151.328	0.453	0.975	0.0452

**Table 4:** Adsorption isotherm analysis of FS-N-2M-2 h-293K.

Temperature (K)	Langmuir Isotherm Model				
	K_L_	Q_max_	R^2^	ALL	Equation
293	0.321	320.134	0.982	0.135	Q_e_ = 102.808C_e_/(1 + 0.321C_e_)
303	0.244	308.616	0.916	0.170	Q_e_ = 75.173C_e_/(1 + 0.244C_e_)
313	0.167	287.880	0.955	0.230	Q_e_ = 48.145C_e_/(1 + 0.167C_e_)
323	0.146	273.099	0.939	0.256	Q_e_ = 39.782C_e_/(1 + 0.146C_e_)
**Temperature** **(K)**	**Freundlich Isotherm Model**				
	**K_F_**	**n**	**R^2^**	**Equation**	
293	127.085	4.881	0.879	Q_e_ = 127.085C_e_^0.205^	
303	107.450	4.332	0.838	Q_e_ = 107.45C_e_^0.231^	
313	100.253	4.654	0.757	Q_e_ = 100.253C_e_^0.215^	
323	92.136	4.591	0.748	Q_e_ = 92.136C_e_^0.218^	
**Temperature** **(K)**	**Temkin Isotherm Model**				
	**K_T_**	**b**	**R^2^**	**Equation**	
293	52.087	10.268	0.952	Q_e_ = 237.24ln (52.087C_e_)	
303	47.291	4.179	0.901	Q_e_ = 781.843ln (47.291C_e_)	
313	50.494	3.025	0.854	Q_e_ = 860.313ln (50.494C_e_)	
323	52.595	2.567	0.839	Q_e_ = 1046.271ln (52.595C_e_)	

**Table 5:** Thermodynamic analysis of FS-N-2M-2 h-293K.

Δ*G*(kJ/mol)				Δ*S*	Δ*H*	R^2^
293 K	303 K	313 K	323 K	(J/(mol·K))	(kJ/mol)	
−4.21	−3.936	−3.905	−3.825	−82.398	−37.526	0.9366

**Table 6:** Performance comparison of adsorption effect.

Adsorbent	Qm (mg/g)	Equilibrum Time (min)	pH	T (K)	References
Carbon nanotubes	65.36	120	9.4	293	[51]
Clay	88	-	-	Room temperature	[52]
hydrocarbon textile waste	72	90	10	Room temperature	[53]
tea cellulose hydrogel	41.67	300	10	298	[54]
magnetic graphene oxide	205.34	120	10	318	[55]
FS-N-2M-2 h-293K	162.94	120	9	293	This paper

## Data Availability

The data presented in this study are available on request from the corresponding author.

## Supplementary Materials

The following supporting information can be downloaded at: https://www.mdpi.com/article/10.3390/ijerph191912851/s1, Figure S1. Acid concentration on the leaching rate of metal: Ca-leaching changes of calcium; Al-leaching changes of aluminum; Fe-iron leaching change; Si-silicon leaching changes. Figure S2. Leaching time on the leaching efficiency of metal: Ca-leaching changes of calcium; Al-leaching changes of aluminum; Fe-leaching change of iron; Si-leaching changes of silicon. Figure S3. Temperature on the leaching efficiency of metal: Ca-leaching changes of calcium; Al-leaching changes of aluminum; Fe-leaching change of iron; Si-leaching changes of silicon. Figure S4. XRD analysis of initial FS and functional adsorbents leached by acid: (a) initial FS, (b) HNO_3_, (c) HCl, (d) H_2_SO_4_, (e) HAc, (f) HF. Figure S5. The SEM-EDS analysis of initial FS and functional adsorbents: (a) initial FS, (b) FS-N-2M-2 h-293K, (c) FS-H-2M-2 h-293K. Figure S6. The SEM-EDS analysis of functional adsorbents: (a) FS-S-2M-2 h-293, (b) FS-F-2M-2 h-293K, (c) FS-C-2M-2 h-293K. Table S1. Design and results of orthogonal experiment. Table S2. Correlation analysis of leaching rate and adsorption capacity. Table S3. Multifactor parameter analysis of leaching efficiency (R_LE_) and adsorption capacity (C_AC_). Table S4. Design and results of orthogonal experiment. Table S5. Orthogonal experimental analysis of adsorption capacity.

## Abbreviations

CGS	Coal gasification slag
FS	Coal gasification fine slag
CS	Coal gasification coarse slag
MB	Methylene blue
*R_LE_*	Leaching efficiency
C_AC_	Adsorption capacity
FS-N-2M-2 h-293K	The sample was obtained by 2.0 mol/L HNO_3_ at 293 K for 2 h.
FS-H-2M-2 h-293K	The sample was obtained by 2.0 mol/L HCl at 293 K for 2 h.
FS-S-2M-2 h-293K	The sample was obtained by 2.0 mol/L H_2_SO_4_ at 293 K for 2 h.
FS-F-2M-2 h-293K	The sample was obtained by 2.0 mol/L HF at 293 K for 2 h.
FS-C-2M-2 h-293K	The sample was obtained by 2.0 mol/L HAc at 293 K for 2 h.
