# Supplementary material for: Using One-Step Acid Leaching for the Recovering of Coal Gasification Fine Slag as Functional Adsorbents: Preparation and Performance
Source: Int J Environ Res Public Health. 2022 Oct 7;19(19):12851. doi: 10.3390/ijerph191912851 (PMC9564584; doi:10.3390/ijerph191912851)
Supplement: Supplementary file 1 [file ijerph-19-12851-s001.zip › ijerph-1887573-supplementary.pdf]

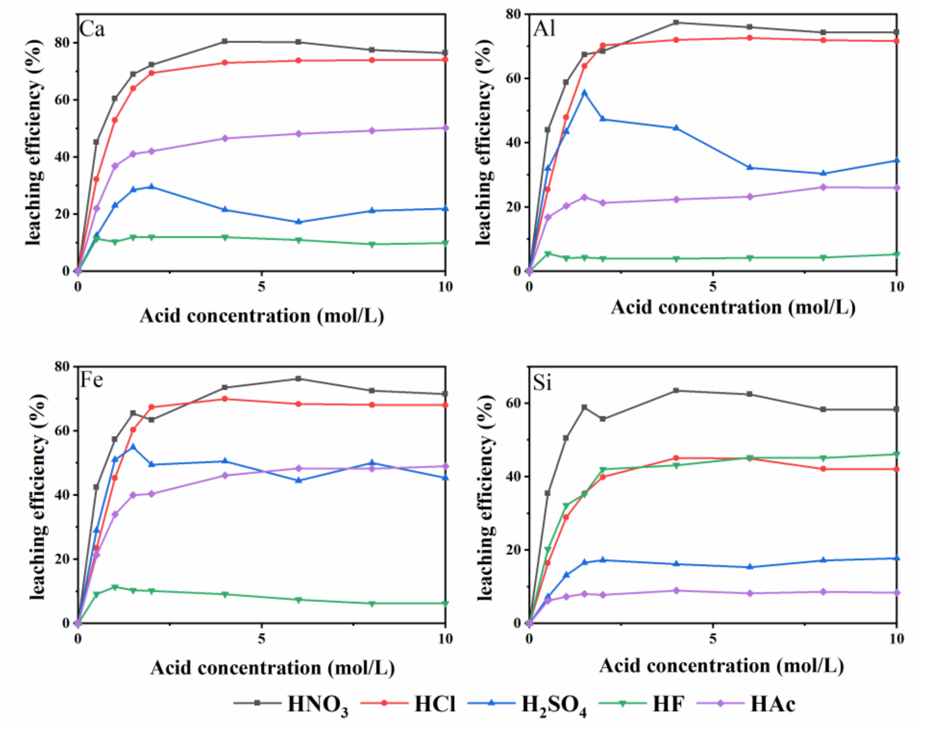

Figure S1. Acid concentration on the leaching efficiency of metal: Ca-leaching changes of calcium; Al-leaching changes of aluminum; Fe-leaching change of iron; Si-leaching changes of silicon.

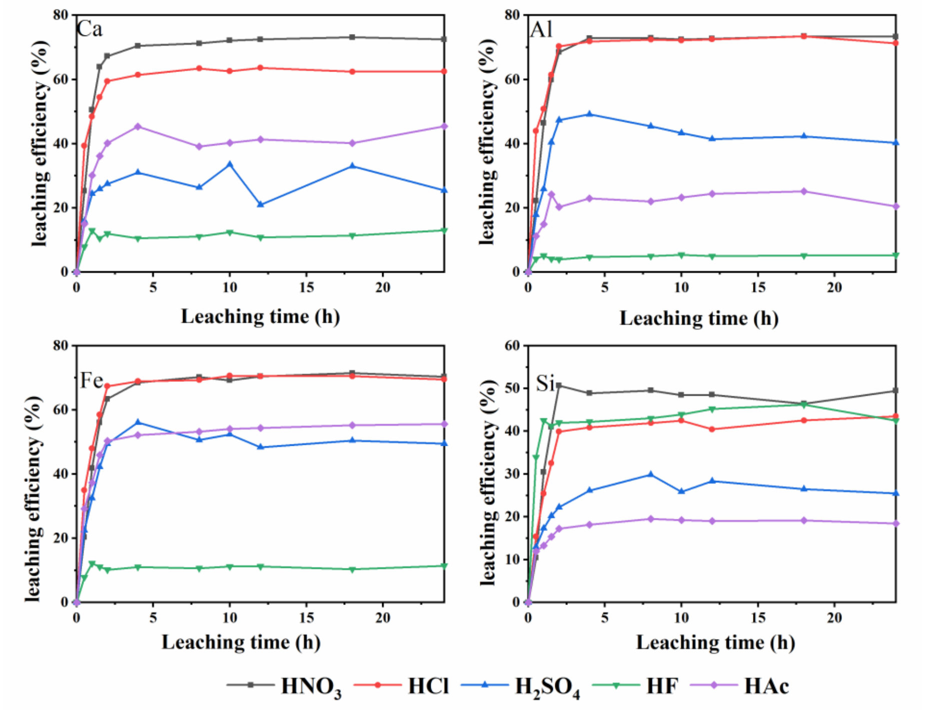

Figure S2. Leaching time on the leaching efficiency of metal: Ca-leaching changes of calcium; Al-leaching changes of aluminum; Fe-leaching change of iron ; Si-leaching changes of silicon.

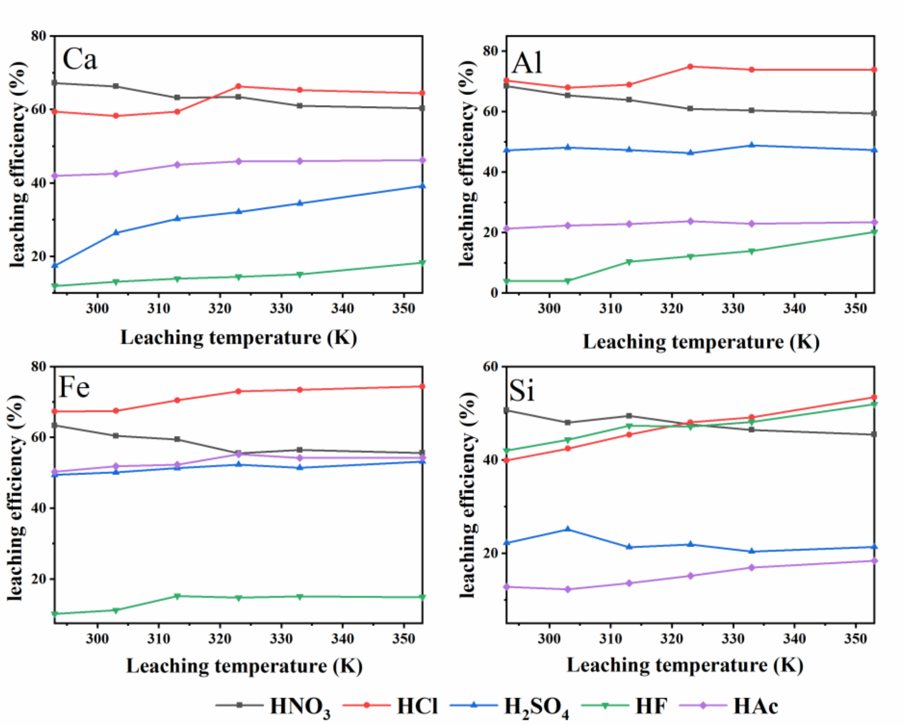

Figure S3. Temperature on the leaching efficiency of metal: Ca-leaching changes of calcium; Al-leaching changes of aluminum; Fe-leaching change of iron; Si-leaching changes of silicon.

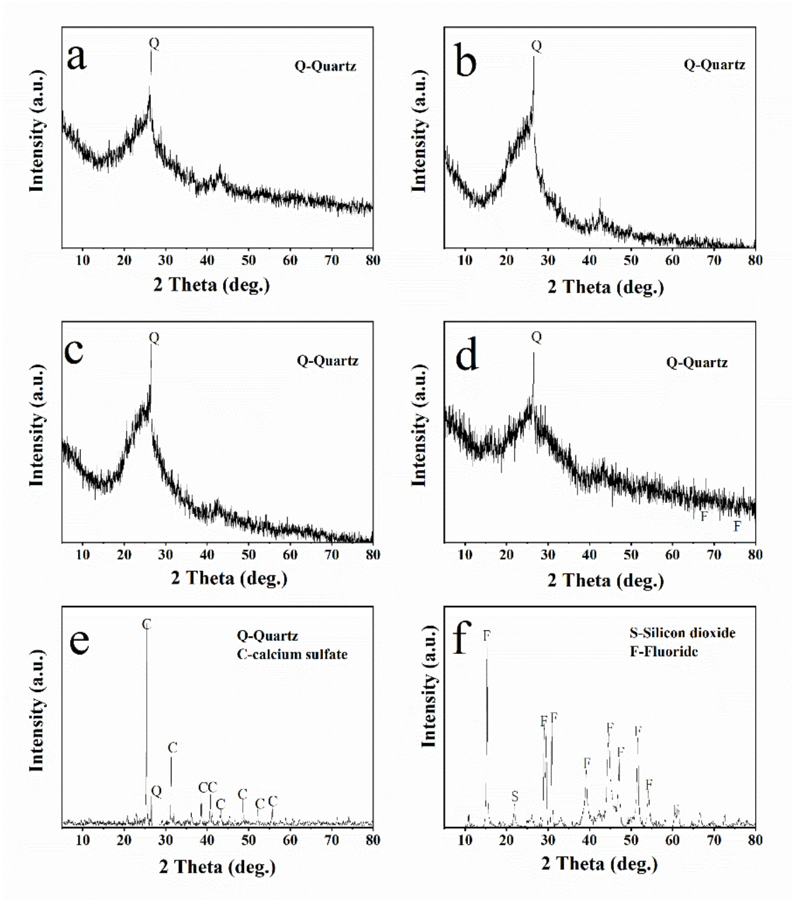

Figure S4. XRD analysis of initial FS and functional adsorbents leached by acid: (**a**) initial FS, (**b**) HNO_3, (**c**) HCl, (**d**) H_2SO_4, (**e**) HAc, (**f**) HF.

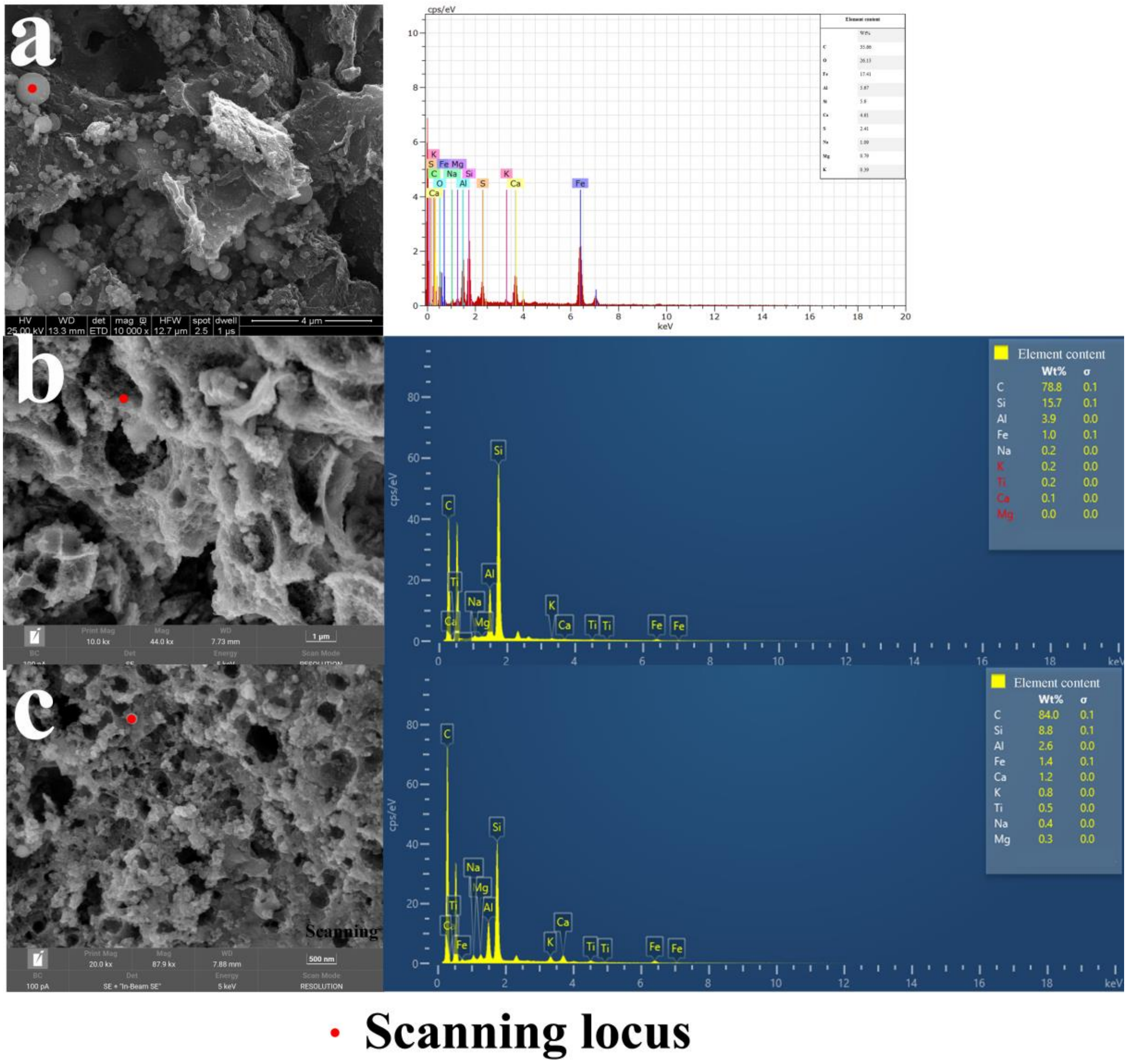

Figure S5. The SEM-EDS analysis of initial FS and functional adsorbents: (a) initial FS, (b) FS-N-2M-2h-293K, (c) FS-H-2M-2h-293K.

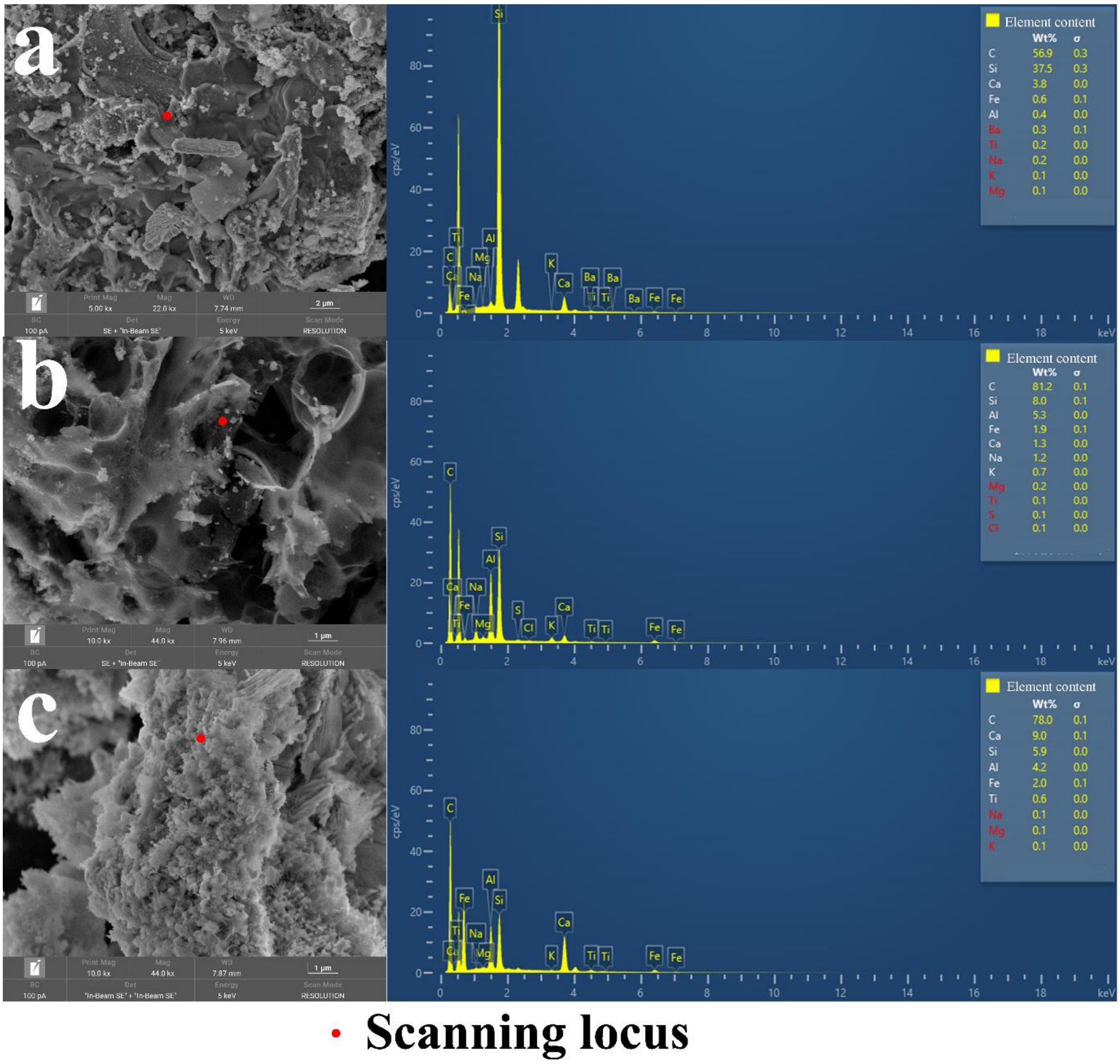

Figure S6. The SEM-EDS analysis of functional adsorbents: (a) FS-S-2M-2h-293, (b) FS-F-2M-2h-293K, (c) FS-C-2M-2h-293K.

Table S1. Design and results of orthogonal experiment.

Acid type	Leaching temperature	Leaching Time	Acid concentration	Efficiency of leaching	Capacity of adsorbing
HNO_3	293	0.5	0.5	0.31351	121.3185
HNO_3	303	1	1	0.326589	130.8755
HNO_3	313	1.5	1.5	0.451087	144.2135
HNO_3	323	2	2	0.5743	175.7235
HCl	293	1	1.5	0.331247	112.2315
HCl	303	0.5	2	0.256983	106.8319
HCl	313	2	0.5	0.334982	114.1878
HCl	323	1.5	1	0.321236	120.5435
H_2SO_4	293	1.5	2	0.31531	109.5369
H_2SO_4	303	2	1.5	0.335212	112.1284
H_2SO_4	313	0.5	1	0.169482	98.19849
H_2SO_4	323	1	0.5	0.250984	90.56218
HAc	293	2	1	0.272352	82.18756
HAc	303	1.5	0.5	0.213543	80.19329
HAc	313	1	2	0.359893	87.21939
HAc	323	0.5	1.5	0.168876	80.13799

Table S2. Correlation analysis of leaching efficiency and adsorption capacity.

		Acid concentration		Leaching time		Leaching temperature	
		Coefficient	Sig.	Coefficient	Sig.	Coefficient	Sig.
Pearson	HNO_3	0.978	0	0.988	0	0.851	0.032
	HCl	0.913	0.001	0.857	0.001	0.848	0.033
	H_2SO_4	0.343	0.367	0.857	0.001	0.839	0.037
	HF	-0.309	0.418	-0.037	0.914	0.646	0.166
	HAc	0.913	0.001	0.904	0	-0.41	0.42
	Total	0.618	0	0.575	0	0.87	0
Kendall τ	HNO_3	0.778	0.004	0.6	0.1	0.733	0.039
	HCl	1	0	0.273	0.243	0.6	0.091
	H_2SO_4	0.5	0.061	0.345	0.139	0.867	0.015
	HF	0	1	0.018	0.938	0.733	0.039
	HAc	0.889	0.001	0.818	0	-0.2	0.573
	Total	0.573	0	0.471	0	0.687	0

Table S3. Multifactor parameter analysis of leaching efficiency (R_{LE}) and adsorption capacity (C_{AC}).

		R^2	Type III Sum of Squares			
			acid concentration	Leaching Temperature	leaching Time	acid type
HNO_3	R_{LE}	0.992	0.352	0.002	0.333	
	C_{AC}	0.986	9058.661	258.486	8680.57	
HCl	R_{LE}	0.999	0.238	0.019	0.236	
	C_{AC}	0.962	3319.169	739.312	3198.04	
H_2SO_4	R_{LE}	0.985	0.188	0.011	0.257	
	C_{AC}	0.988	4420.79	1867.38	1248.49	
HF	R_{LE}	0.985	0.189	0.002	0.162	
	C_{AC}	0.885	1283.838	709.172	407.847	
HAc	R_{LE}	0.993	0.286	0.003	0.271	
	C_{AC}	0.958	598.71	381.074	873.011	
Total	R_{LE}	0.991	1.17	0.002	1.32	0.19
	C_{AC}	0.991	6608.968	610.506	7570.37	36513.9

Table S4. Design and results of orthogonal experiment.

Level	Acid type	Leaching temperature	Leaching time	Acid Concentration
1	0.4164	0.3081	0.2272	0.2783
2	0.3111	0.2831	0.3172	0.2724
3	0.2677	0.3289	0.3253	0.3216
4	0.2537	0.3288	0.3792	0.3766
Delta	0.1627	0.0458	0.152	0.1042
Rank	1	4	2	3

Table S5. Orthogonal experimental analysis of adsorption capacity.

Level	Acid type	Leaching temperature	Leaching time	Acid Concentration
1	143.03	106.32	101.62	101.57
2	113.45	107.51	105.22	107.95
3	102.61	110.95	113.62	112.18
4	82.43	116.74	121.06	119.83
Delta	60.6	10.42	19.44	18.26
Rank	1	4	2	3
